# A Comparison of Various Chips Used for the Manufacture of Biosensors Applied in Non-Fluidic Array SPRi, Based on the Example of Determination of Cathepsin D

**DOI:** 10.3390/bios12010021

**Published:** 2021-12-31

**Authors:** Pawel Falkowski, Piotr Mrozek, Piotr Miluski, Zenon Lukaszewski, Ewa Gorodkiewicz

**Affiliations:** 1 Bioanalysis Laboratory, Faculty of Chemistry, University of Bialystok, Ciolkowskiego 1K, 15-245 Bialystok, Poland; pawelfalkowski@wp.pl; 2 Faculty of Mechanical Engineering, Bialystok University of Technology, Wiejska 45 C, 15-351 Bialystok, Poland; p.mrozek@pb.edu.pl; 3 Faculty of Electrical Engineering, Bialystok University of Technology, Wiejska 45 C, 15-351 Bialystok, Poland; p.miluski@pb.edu.pl; 4 Faculty of Chemical Technology, Poznan University of Technology, Sklodowskiej-Curie 5, 60-965 Poznan, Poland

**Keywords:** cathepsin D, array SPRi, Ag/Au chip, blood serum, adhesive separating foil, glioblastoma

## Abstract

Non-fluidic array SPR imaging (SPRi) with appropriate biosensors is a new tool for the determination of various biomarkers in body fluids. Numerous biomarkers can be determined without signal enhancement or preliminarily preconcentration. The introduction of a new material solution of the chip may increase the scope of the application of this technique. Solutions with adhesive separating foil and an Ag/Au chip were compared with the previously used two-paint separating polymer and pure gold chip. These solutions were tested using the example of a biosensor for cathepsin D (Cath D), which consisted of pepstatin A (a Cath D inhibitor) immobilized via a cysteamine linker using the NHS/EDC protocol. Four material versions of the Cath D biosensor proved adequate in terms of range of linearity, LOQ, precision and recovery. All four versions of the biosensor were used for the determination of Cath D in the blood serum patients with glioblastoma and control samples, producing very similar results and showing an elevated biomarker concentration in the case of cancer. Therefore, the problem of determining the correct level of Cath D in the serum of healthy individuals has been resolved, correcting literature data which ranged over three orders of magnitude.

## 1. Introduction

Array SPR imaging (SPRi) of appropriate biosensors is well suited to the determination of various biomarkers in body fluids. Biosensors have been developed for the determination of the biomarkers HE 4 [1], CA 125 [2], CEA [3], leptin [4], MMP-1, MMP-2, laminin-5, fibronectin, collagen IV, UCHL-1, proteasome 20S and immunoproteasome 20S, aromatase, podoplanin, cathepsins B, D, G, L, and S, and cystatin C, in body fluids. Most of these are described, among other biosensors, in two review papers [5,6]. The majority of these biosensors have been used in clinical investigations, including studies concerning bladder cancer [7,8,9], endometriosis [6,7,8,9,10], burns healing [11], acute appendicitis [12], and cryptorchidism [13]. In contrast to the fluidic SPR technique, these biosensors are suitable for the determination of a required marker without signal enhancement or preliminary preconcentration, which significantly facilitates the determination of a biomarker in a body fluid such as human serum. In order to achieve sufficiently low biomarker concentration, the commonly used fluidic SPR requires strong signal enhancement, for example, by the formation of a sandwich structure in a biosensor containing gold nanoparticles [14,15]. The two main differences between array SPRi and fluidic SPR lie in the sequence of biosensor formation and the presence or absence of water solution during SPR measurement. In array SPRi, a biosensor is formed ex situ, prior to measurement, whereas in fluidic SPR it is formed in situ during measurement. The water solution is gently removed prior to measurement in array SPRi, whereas fluidic SPR measurement is performed in the presence of water solution. Another advantage of array SPRi is the relatively simple biosensor structure: it consists of an antibody against a biomarker, or an inhibitor of the biomarker immobilized via a suitable linker. Thus, array SPRi is a label-free method. The biosensor structure in the array SPRi technique is significantly simpler than that of the commonly used ELISA immunosensors; the latter is also a label-containing method.

Array SPRi uses chips, having an array of measuring points on the chip surface. The measuring points are separated by polymers. Usually, an array of approximately one hundred measuring points is used. Prior to the formation of the biosensor, bare gold is present on the bottom of a measuring point. The measuring points form several measuring cells separated by hydrophobic paint, which enable the measurement of several samples simultaneously. Each measuring cell contains about a dozen measuring points. The average result from the measuring points of a cell is considered as a single result. Apart from gold chips, chips based on silver covered with a thick gold layer have recently been used for biosensor preparation, exhibiting slightly better analytical characteristics than a biosensor based on gold chips [16,17]. The manufacture of polymer layers containing two paints is a relatively complex technological process. However, the manufactured chips are regenerable, and can be used up to ten times after a suitable cleaning process [18]. Due to the relative complexity of the chip manufacturing process described above, the use of adhesive polymeric foil has been proposed to simplify chip manufacturing. The aim of this study was to investigate this possibility, and to compare four different chips in terms of the analytical characteristics of a selected biosensor. These four chips represented different material solutions: (i) an Au chip covered with two paints; (ii) an Au chip covered with adhesive polymeric foil; (iii) an Ag/Au chip covered with two paints; and (iv) an Ag/Au chip covered with adhesive polymeric foil. If all four chips exhibited satisfactory analytical characteristics, the competitive potential of the non-fluidic array SPRi technique would be significantly enhanced.

A biosensor for the determination of cathepsin D (Cath D), made using the four above-mentioned chips, was selected for this investigation. Cath D is a significant biomarker of Alzheimer’s disease [19,20], neurodegenerative diseases [21], breast cancer [22], coronary heart disease [23] and type 2 diabetes [24]. It is a soluble lysosomal aspartic acidic endopeptidase. Cath D exhibits optimum activity below pH 5 [25], and its MW is 48 kDa. Recently reported values of Cath D concentration in human serum range over four orders of magnitude, from 0.144 to 1690 ng mL^−1^ (see Table 1).

The concentration of a marker may be elevated in case of disease. However, the level of a biomarker in the serum of a healthy person (control) exhibits relatively low variation, of a maximum of one order of magnitude. Therefore, the variation of Cath D in the serum of healthy subjects over three orders of magnitude (from 0.144 to 123.6 ng mL^−1^, see Table 1) indicates a very significant analytical problem, and casts doubt on the credibility of all of the reported results. These results were obtained with ELISA immunoassays from different manufacturers. Assuming that there were no procedural errors in these studies, the conclusion was that ELISA immunoassays are not a reliable tool for the determination of Cath D. Despite the general similarity of ELISA immunoassays, the particular solutions applied may vary: the primary and secondary antibodies used may be different. It should be noted here that the secondary antibody was conjugated with horseradish peroxidase, and used as a label.

Generally, an independent method is required to resolve the problem of which immunoassay provides correct measurements. Such an opportunity is provided by an SPRi biosensor and its related method [33]. The SPRi biosensor uses pepstatin A as a receptor, selectively bonding Cath D. The receptor was immobilized using the NHS/EDS protocol via a cysteamine linker. Selectivity of the biosensor was checked in the presence of cathepsin B (1:1000). Thus, an additional aim of this study was to provide diagnostics using a new tool for the determination of Cath D in blood serum.

## 2. Materials and Methods

### 2.1. SPRi Apparatus

SPRi measurements were performed on a prototype apparatus developed at the University of Bialystok, which has been described in a previous paper [17]. Briefly, the apparatus uses the Kretchmann configuration, a diode laser (635 nm), a linear polarizer and CCD digital camera; “p” polarization was used for basic measurements, and “s” polarization for measurement of the background.

### 2.2. Chip Manufacture

Chips covered with a layer of gold (50 nm) were supplied by Sens (Ssens BV, Enschede, The Netherlands). Chips covered with a bimetallic layer of silver and gold were manufactured as described in the previous paper [17]. Briefly, thin metallic films were deposited onto the surface of the glass by means of physical vapor deposition in a vacuum system at ambient temperature. The film consisted of a 1 nm chromium adhesive layer, 42 nm of silver and 5 nm of gold.

### 2.3. Preparation of Separating Paint Layer

Separating paint layers were prepared as described in the previous paper [17]. Briefly, two paint layers were formed successively on both Ag/Au and pure gold chips using a screen-printing technique. The first layer covered all of the chip surface, with the exception of 108 measuring points exposing the pure gold surface. The second layer, being hydrophobic, separated nine measuring cells on the chip surface.

### 2.4. Preparation of Adhesive Polymeric Foil Layer

**Figure 1:**
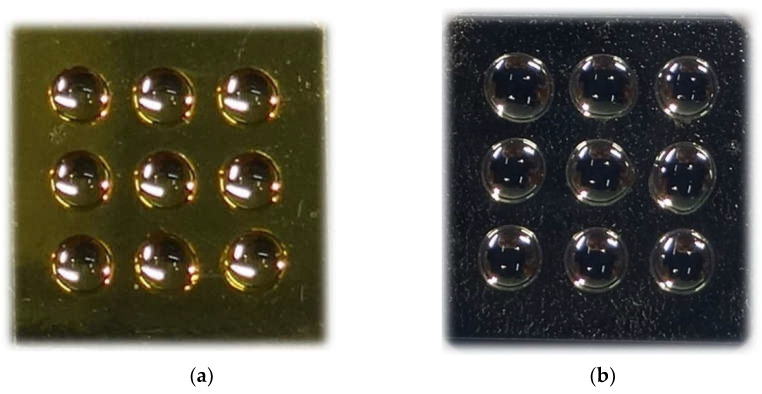
Photograph showing chip made from (**a**) pure gold; (**b**) Ag/Au covered with adhesive foil and nine measured samples.

The masks used to determine the distribution of measurement fields on the surface of the biosensor were made from a thin polypropylene self-adhesive foil, using the punching method. A numerically controlled device was designed and manufactured, equipped with a holder enabling the installation of a circular-shaped die, with the diameter of the working part selected according to the requirements of the sensor’s sensory field design. The dies used enable holes to be made in the polymer foil with diameters ranging from a few tenths of a millimetre to a few millimetres. The number and spacing of the holes punched in the mask were configured in the computer program controlling the device. The pressure force of the die was selected according to its diameter and the thickness of the foil used. The automated method of making holes in the mask guaranteed the precision of their spacing and ensured the repeatability of mask manufacture. The hydrophobic properties of the mask material maintained the separation of individual drops of analyte solution applied to adjacent measurement fields of the mask deposited on the sensor surface during sensor operation (see Figure 1).

### 2.5. Reagents

The following reagents were used in the experimental part: Cathepsin D protein (SIGMA, Steinheim, Germany) and its specific inhibitor—pepstatin A (SIGMA, Steinheim, Germany), human albumine, (SIGMA, Steinheim, Germany), *N*-hydroxysuccinimide (NHS) (SIGMA ALDRICH, Munich, Germany), *N*-Ethyl-*N′*-(3-dimethylaminopropyl) carbodiimide (EDC) (SIGMA ALDRICH, Munich, Germany), cysteamine hydrochloride acting as a linker (SIGMA ALDRICH, Munich, Germany), phosphate-buffered saline (PBS) pH = 7.4 (BIOMED, Lublin, Poland), acetate buffer pH = 3.50 (BIOMED, Lublin, Poland), carbonate buffer pH = 8.50–9.86 (BIOMED, Lublin, Poland).

The solvents used to prepare solutions were absolute ethanol 99.8% (POCh, Gliwice, Poland) and MilliQ water (Simplicity^®^ Millipore). Measurements were performed with two types of glass chips coated with ultrathin metal layers: the first with a 50 nm layer of gold (Sens, Netherlands), and the second evaporated with a 42 nm layer of silver and a 5 nm layer of gold (Bialystok University of Technology).

### 2.6. Biological Material

For the purposes of this study, a biological plasma material was used. Samples were collected from patients with glioblastoma (stage IV). Prior to analysis, the samples were prepared by 10-fold dilution with an acetate buffer, pH = 3.50 (BIOMED, Lublin, Poland). Control probes were taken from a group of smokers and were prepared by 5-fold dilution with an acetate buffer pH = 3.50 (BIOMED, Lublin, Poland).

All samples came from the Biobank of the Medical University in Bialystok (Bioethics Committee approval No.002.171.2021).

### 2.7. Procedure for Biosensor Preparation

The procedure for sensor preparation consisted of two steps: the formation of a cysteamine monolayer, and immobilization of the inhibitor. In the first step, glass chips covered with a metal layer were washed five times with MilliQ water and absolute ethyl alcohol, and then dried under a stream of inert gas (argon). Pure and dried glass chips were then immersed in a 20 mM alcohol solution of cysteamine hydrochloride for 12 h in a glass jar at room temperature. After this time, the chip was rinsed 10 times, with MilliQ water and absolute ethanol, alternately. The residual solvent was removed under a stream of argon.

In the second step, pepstatin A (an inhibitor) was activated by mixing with N-hydroxysuccinimide (250 nM) and N-Ethyl-N’-(3-dimethylaminopropyl) carbodiimide (250 nM) in the presence of carbonate buffer (pH = 8.5). After 5 min of mixing, the activated solution of pepstatin A was applied to the active areas of a chip in the form of small droplets (3.5 uL), and was then stored in an oven for 1 h for final bonding. After this time, the pepstatin A solution was removed by rinsing with ethanol and MilliQ water at least 10 times, and the chip was dried in an atmosphere of argon.

### 2.8. SPRi Measurement

SPRi measurements were performed using a dedicated device described in previous papers [17]. The measurements were performed in two steps: (1) the determination of the SPR angle at the highest drop of iridescent light intensity; (2) recording of the chip image scans in two polarization modes—bright (p) and dark (s)—for chips with and without cathepsin D. First, a determination was made of the SPR angle at which the contrast of light intensity between the measuring cells and the background gave the highest value. In the second step, sensor scan images were collected to provide data for further analysis. For this purpose, all images were recorded in two polarization modes—bright (p) and dark (s)—for the pure immobilized sensor without analyte (cathepsin D). The same procedure was repeated for sensors with applied cathepsin D. The principle underlying the whole procedure was the SPR phenomenon. Adsorption of cathepsin D influenced the interface characteristics, causing a decrease in light intensity, and this decrease corresponded to the cathepsin D concentration. Thus, the same recording procedure was used for sensors before and after interaction with cathepsin D.

## 3. Results

Analytical characteristics of biosensors for the determination of Cath D formed on pure gold and Ag/Au chips covered with different polymeric materials.

**Figure 2:**
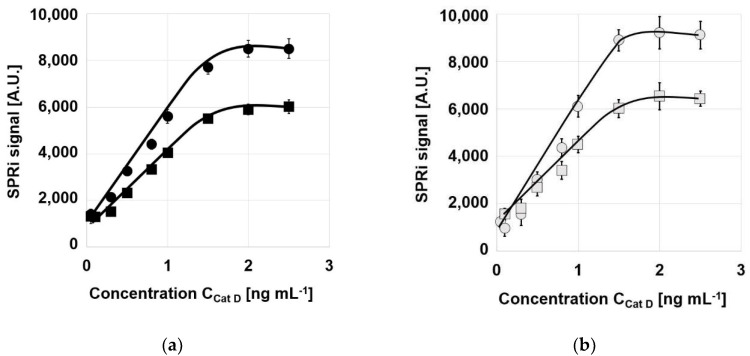
Calibration graphs of Cath D biosensors based on: (**a**) pure gold chips; (**b**) Ag/Au chips, covered with adhesive foil (circles) or two photopaints (squares). Concentration of pepstatin A: 500 ng mL^−1^, pH: 3.75.

Typical analytical characteristics of the biosensors—calibration graphs, linearity range, precision, recovery, and limits of detection and quantification—were investigated. The concentration of pepstatin A used as a receptor was selected as 500 ng mL^−1^, based on a study by Gorodkiewicz and Regulska [33], and the optimum pH was taken as 3.75. Calibration graphs were investigated within the range 0.1–2.5 ng mL^−1^. The results are shown in Figure 2 for the biosensors based on pure gold (Figure 2a) and on Ag/Au chips (Figure 2b). Typical Langmuirian curves were obtained, with a plateau and an approximately linear section. Slight differences are visible between the biosensors based on chips covered with adhesive foil (circles) and those covered with two photopaints (squares).

**Figure 3:**
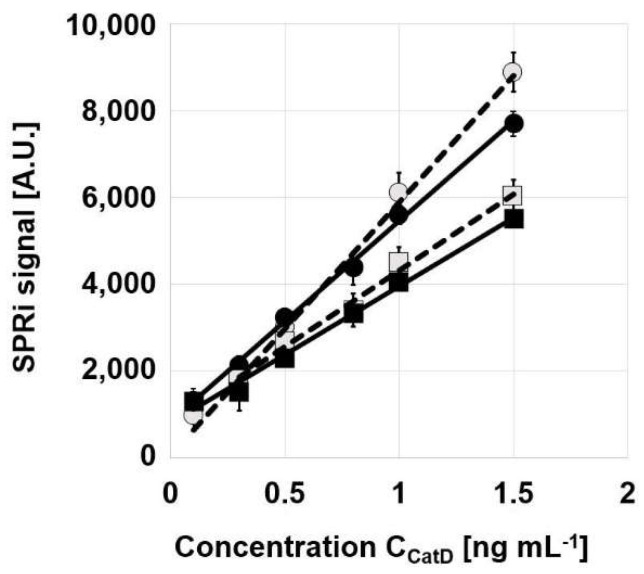
Linear sections of calibration graphs for the determination of Cath D. Pure gold chips, squares; Ag/Au chips, circles; black points, two paints; open points, adhesive foil. Concentration of pepstatin A: 500 ng mL^−1^, pH: 3.75.

The initial sections of these graphs are shown in Figure 3. All four curves fit well to straight lines. The parameters of these lines are summarized in Table 2.

All four curves exhibit linearity within the range 0.1–1.5 ng mL^−1^. The lower accessible concentration will be corrected to the value of the LOQ. All four calibration graphs show good linear fits, with R^2^ above 0.99. The slopes of the graphs differ: the calibration curves measured using the biosensors based on Ag/Au chips have higher slopes than those based on pure gold chips, and the curves measured using the biosensors with adhesive foil have higher slopes than those with two-paint polymer separation.

Four versions of the biosensor for the determination of Cath D were investigated in terms of precision of measurement and recovery. Each version was investigated for three levels of Cath D concentration. The results are shown in four tables: Table 3 for the biosensor based on a pure gold chip with two-paint polymer separation; Table 4 for the biosensor based on a pure gold chip with adhesive foil as separating polymer; Table 5 for the biosensor based on an Ag/Au chip with two-paint polymer separation; Table 6 for the biosensor based on an Ag/Au chip with adhesive foil as separating polymer.

All four versions of the biosensor for Cath D show acceptable recoveries, generally between 95% and 108%, with the exception of the lowest concentration in the case of the biosensor containing an Ag/Au chip and adhesive foil (Table 6. The precision of measurement for the biosensors with adhesive foil is significantly worse than that for the biosensors with two-paint separation, with the best precision obtained for the biosensor containing an Ag/Au chip and two paints. The limits of quantification, which represent the lowest Cath D concentrations quantifiable with the use of particular versions of the biosensor, range from 0.17 ng mL^−1^ to 0.40 ng mL^−1^, and better values were obtained for the biosensors with adhesive foil separation. Therefore, the accessible range of Cath D concentration is equal to LOQ-1.5 ng mL^−1^ and is narrower than that shown in Figure 3.

The investigation of analytical characteristics under the model conditions described above provided only a preliminary evaluation of the potential of biosensors for the determination of Cath D based on different chips and separating polymers. The final evaluation of this potential required the determination of Cath D in real samples. Therefore, Cath D was determined in nine samples of serum from a control group and nine samples of serum from patients with glioblastoma. The Cath D concentration was determined using the four versions of the biosensor described above. The results are shown in Table 7 and Table 8. Serum samples were suitably diluted with acetate buffer (pH 3.5) to bring the concentration within the linear section of the calibration graph.

## 4. Discussion

A significant question arises of whether the results obtained with the four versions of the biosensor for Cath D determination are equivalent. In the series of results for serum from control group, those obtained using the biosensor based on an Ag/Au chip with adhesive foil as a separating polymer were generally higher than those obtained with the other versions of the biosensor, whereas the results obtained with the biosensor based on a pure gold chip with adhesive foil were the lowest. The difference between the maximum and minimum results was equal to 12.5%, on average. These differences were smaller in the case of the other two versions of the biosensor.

The results of Cath D concentration obtained with the biosensor formed on the pure gold chip with the adhesive foil are the highest (see Table 8 in the case of the serum of the glioblastoma patients, opposite than in the case of the serum of healthy volunteers. The lowest results in this series were obtained for the biosensor formed on the Ag/Au chip and the two-paint separating polymer layer (see Table 8 )). The difference between the highest and the lowest results was 11.3%. The conclusion from these considerations is that the results for Cath D determination using four versions of the biosensor were equivalent.

The difference in the slopes of the straight sections of the calibration graphs (see Table 2) has no real practical significance for the determination of Cath D in serum. Cath D serum concentration exceeded the range of dynamic response for all curves, and samples had to be adequately diluted with acetate buffer (pH 3.5) to fit the corresponding calibration curve. Thus, there is no reason to conclude in this case that the biosensor whose calibration graph had the highest slope (that based on Ag/Au with adhesive foil) was the best. However, in the determination of other analytes with lower concentrations in blood, the higher slope for the biosensor based on Ag/Au with adhesive foil may be significant. It is worth mentioning that the range of linearity can be extended by using the logarithm of concentration vs. the SPRi signal, instead of the concentration vs. the SPRi signal.

Surprisingly, the precision of Cath D determination in serum samples was better than in the model investigations (compare Table 3, Table 4, Table 5 and Table 6 with Table 7 and Table 8). Only a few results in Table 4, and none in Table 5, had a precision exceeding 10%. None of the biosensor versions differed from the others in terms of precision. This situation was better than in the case of the results for the two biosensors formed using adhesive foil (see Table 4 and Table 6), where the precision of measurement was significantly worse. The authors are not able to explain these differences at present.

The question arises of which version of the biosensor for the determination of Cath D should be recommended to potential users. All four versions were equivalent in terms of analytical characteristics. The versions using pure gold chips provided the possibility of purchasing commercially available, well-standardized chips. The application of adhesive foil seems to be technically the simplest method of preparation of the biosensor. However, such a biosensor is not regenerable. The use of a gold chip for just nine measurements is the most expensive of the four options. The gold chip covered with two paint layers is a technically more difficult version; however, the product is regenerable. Up to ten measurements can be performed using this basis for the biosensor, and 90 measurements can be performed using a single chip. This reduces the cost per single measurement. Ag/Au chips are not yet commercially available, but should be potentially cheaper than pure gold chips, due to the ratio of gold and silver prices. Therefore, disposable Ag/Au chips with adhesive separating foil may be an acceptable version of the biosensor from the point of view of the cost per single measurement. To date, the authors have no knowledge about the possibility of regeneration of Ag/Au chips covered with the two-paint separating layers. Such a solution appears the most complicated, due to the need to produce both the Ag/Au chip and the two paint layers. This may be verified in future commercial evaluations. The developed biosensors are stable during a single day. However, the chip with the cysteamine layer is stable for one month.

This paper provides significant arguments in the discussion concerning the correct level of Cath D in human serum, and which ELISA immunosensors give credible results. In the comparison of the results obtained with various immunosensors, only those for healthy persons were taken into account, because different diseases can significantly affect the level of Cath D in the serum (see Table 1). Reported results for Cath D in blood serum varied from 0.144–0.46 [26] to 126.6 ng mL^−1^ [31] with a median of 4.22 ng mL^−1^ [26,27], that is, they ranged over more than three orders of magnitude. Therefore, the results of the present study, which give an average value of 2.88 ± 0.6 ng mL^−1^, are in good agreement with the median of the results reported in the literature (4.22 ± 3.01; 4.20 ± 1.65 ng mL^−1^). However, the results of this study are not entirely representative for Cath D serum levels, because only nine serum samples were examined and, additionally, they all came from persons over 50 years of age. Concluding, the median range of 4.22 ± 3.01 ng mL^−1^ [29] determined on the basis of 22 results for Korean healthy individuals, includes the averages for 40 Turkish healthy individuals (4.20 ± 1.65 ng mL^−1^) [28], 40 Chinese healthy individuals (2.7 ± 0.8 ng mL^−1^) [27] and 9 Polish individuals (2.88 ± 0.6 ng mL^−1^; this paper), and can be considered to be an established Cath D serum level.

The four versions of the biosensor for determination of Cath D, as described above, may be used as reference methods for the evaluation of available ELISA immunosensors. Unlike all ELISA immunosensors, the SPRi biosensor uses the Cath D inhibitor pepstatin A to entrap the analyte from the tested solution. The inhibitor is immobilized via cysteamine linker on a gold surface using the NHS/EDS protocol. ELISA immunosensors use two antibodies against Cath D: a primary antibody entraps a Cath D molecule, while a secondary antibody, conjugated with horseradish peroxidase (HRP), is entrapped by the entrapped Cath D molecule, forming a sandwich structure. HRP catalyzes the oxidation of the dye by hydrogen peroxide. The last step is a catalytic reaction, and is subject to all of the disadvantages of such reactions. The oxidation of the dye has to be stopped after a certain period of time. The complexity of the ELISA procedure is a source of frequent error. Different antibodies against Cath D may be used for assays produced by different manufacturers. Some of these assays may suffer from insufficient selectivity and give too high results. Thus, the SPRi biosensor, which utilizes a different mechanism of Cath D entrapment, appears to be a suitable reference method for the evaluation of ELISA immunoassays.

The results for Cath D in the serum of patients with glioblastoma were higher than those for the control samples. However, this elevation was hardly above the level of the confidence bars. Therefore, serous Cath D is not a very promising biomarker of glioblastoma. Cath D serum concentration was significantly elevated in cases of other cancers, for example with nasopharyngeal carcinoma (see Table 1) [27], whereas the serous Cath D concentration was almost six times as high as in the control. Such an elevation was not observed in the case of glioblastoma, probably because of the strength of the blood–brain barrier. Without the present work, the fact of the low elevation of Cath D in blood serum over the control would have remained unknown.

## 5. Conclusions

Four versions of a biosensor for the determination of Cath D, representing different material solutions, have been described: (i) an Au chip covered with two separating paints; (ii) an Au chip covered with adhesive polymeric-separating foil; (iii) an Ag/Au chip covered with two separating paints; and (iv), an Ag/Au chip covered with adhesive polymeric-separating foil. All versions exhibited linearity over a concentration range suitable for the determination of Cath D in blood serum, and provided similar precision and recovery. Limits of the quantification ranged from 0.17 ng mL^−1^ to 0.40 ng mL^−1^, and linearity was maintained up to 1.5 ng mL^−1^. Serum samples were adequately diluted with acetic buffer to bring the concentration within the range of linearity. The precision of Cath D determination in serum samples was generally better than 10%, and recoveries were between 95% and 108%. The advantages and disadvantages of each version in terms of manufacture and regenerability have been discussed.

The determination of Cath D in the blood serum of the control group using the described versions of the SPRi biosensor enabled the verification of the correct level of Cath D in serum, for which the data available in the literature give values varying between 0.1 and 126.6 ng mL^−1^. The developed biosensor for Cath D (four versions) served as a suitable reference method, since it used an entirely different method of analyte determination than the other methods. The correct level was determined as equal to the median of available results (4.22 ± 3.01 ng mL^−1^). The results obtained in this study (2.88 ± 0.6 ng mL^−1^) are contained within this range.

The results for Cath D in the serum patients with glioblastoma were higher than those for the control samples (4.85 vs. 2.88 ng mL^−1^ in average).

## Figures and Tables

**Table 1:** Cath D concentration in blood serum.

Disease	Concentration [ng mL^−1^]	Analytical Method	Manufacturer	Reference
Control	0.144–0.46	No data	No data	[26]
Control	2.7 ± 0.8	ELISA	Wuhan Eiaab Science Co. Ltd., Wuhan, China	[27]
Control	4.20 ± 1.65	ELISA	Elabscience, Houston, TX, USA	[28]
Control	4.22 ± 3.01	ELISA	Wuhan USCN, SEB280Hu, China	[19]
Control	12.69 ± 9.29	No data	No data	[29]
Healthy donors	6–15	ELISA	Proteomedix, Switzerland	[30]
Healthy controls	123.6 ± 10.9	ELISA	Abcam, USA	[31]
Control	2.88 ± 0.57	SPRi (a)		This paper
Control	2.73 ± 0.63	SPRi (b)		This paper
Control	2.84 ± 0.63	SPRi (c)		This paper
Control	3.08 ± 0.59	SPRi (d)		This paper
Alzheimer’s	1.836 ± 1.744	ELISA	Wuhan USCN, SEB280Hu, China	[19]
Gaucher	363.1 ± 30.7	ELISA	Abcam, USA	[31]
Nasopharyngeal carcinoma	15.7 ± 8.7	ELISA	Wuhan Eiaab Science Co. Ltd., China	[27]
Type 2 diabetes	234–1690	ELISA	Sincere, Japan	[32]
Preeclampsia	4.97 ± 1.24	ELISA	Elabscience, Houston, USA	[28]
Non-alcoholic fatty liver patients	0.213–0.508	No data	No data	[26]
Glioblastoma	4.71 ± 1.64	SPRi (a)		This paper
Glioblastoma	5.12 ± 1.70	SPRi (b)		This paper
Glioblastoma	4.57 ± 1.62	SPRi (c)		This paper
Glioblastoma	5.02 ± 1.68	SPRi (d)		This paper

(a) Pure gold chip and two-paint separating polymers; (b) pure gold chip and adhesive foil as a separating polymer;.(c) Ag/Au chip and two-paint separating polymers; (d) Ag/Au chip and adhesive foil as a separating polymer.

**Table 2:** Parameters of linear sections of calibration curves of Cath D determination.

Chip	Separating Polymer	Intercept [ng mL^−1^]	Slope [ng mL^−1^]	R^2^
Au	Two paints	793	3152	0.9925
	Foil	827	4618	0.9907
Ag/Au	Two paints	804	3507	0.9978
	Foil	53	5840	0.9915

**Table 3:** Precision and recovery of Cath D determination using the biosensor with a pure gold chip and two-paint separating polymer.

Cath D Spike [ng mL^−1^]	Found [ng mL^−1^]	SD [ng mL^−1^]	RSD [%]	Recovery [%]
0.5	0.47	0.05	10	95
0.8	0.79	0.06	7.5	99
1.0	0.97	0.12	12	97
LOD 0.12				
LOQ 0.40				

**Table 4:** Precision and recovery of Cath D determination using the biosensor with a pure gold chip and adhesive foil as a separating polymer.

Cath D Spike [ng mL^−1^]	Found [ng mL^−1^]	SD [ng mL^−1^]	RSD [%]	Recovery [%]
0.5	0.52	0.09	18	104
0.8	0.77	0.11	14	96
1.0	0.95	0.12	12	95
LOD 0.06				
LOQ 0.20				

**Table 5:** Precision and recovery of Cath D determination using the biosensor with an Ag/Au chip and two-paint separating polymer.

Cath D Spike [ng mL^−1^]	Found [ng mL^−1^]	SD [ng mL^−1^]	RSD [%]	Recovery [%]
0.5	0.52	0.04	8	104
0.8	0.76	0.03	3.8	95
1.0	1.05	0.05	5	105
LOD 0.09				
LOQ 0.30				

**Table 6:** Precision and recovery of Cath D determination using the biosensor with an Ag/Au chip and adhesive foil as a separating polymer.

Cath D Spike [ng mL^−1^]	Found [ng mL^−1^]	SD [ng mL^−1^]	RSD [%]	Recovery [%]
0.3	0.34	0.15	50	113
0.5	0.54	0.08	16	108
0.8	0.80	0.09	11	100
LOD 0.05				
LOQ 0.17				

**Table 7:** Concentration of Cath D in serum of control group, as determined using.

Sample	CathD (a) [ng mL^−1^]	CathD (b) [ng mL^−1^]	CathD (c) [ng mL^−1^]	CathD (d) [ng mL^−1^]
1 2 3 4 5 6 7 8 9	2.93 ± 0.09 3.86 ± 0.12	2.67 ± 0.19 3.25 ± 0.29	2.79 ± 0.12 3.59 ± 0.23	3.03 ± 0.21 4.01 ± 0.24
	2.05 ± 0.24 2.24 ± 0.15 2.40 ± 0.31 3.13 ± 0.18 2.94 ± 0.22 2.90 ± 0.19 3.37 ± 0.17	1.80 ± 0.09 2.02 ± 0.19 2.21 ± 0.09 3.01 ± 0.33 3.21 ± 0.32 2.67 ± 0.23 3.69 ± 0.29	2.01 ± 0.29 2.05 ± 0.17 2.11 ± 0.12 3.31 ± 0.19 3.03 ± 0.21 3.18 ± 0.12 3.49 ± 0.25	2.24 ± 0.36 2.44 ± 0.25 2.60 ± 0.17 3.46 ± 0.25 3.16 ± 0.28 3.10 ± 0.17 3.74 ± 0.31
Average	2.88 ± 0.57	2.73 ± 0.63	2.84 ± 0.63	3.08 ± 0.59

(a) pure gold chip and two-paint separating polymer; (b) pure gold chip and adhesive foil as a separating polymer; (c) Ag/Au chip and two-paint separating polymer; (d) Ag/Au chip and adhesive foil as a separating polymer.

**Table 8:** Concentration of Cath D in serum of patients suffering from glioblastoma as determined using.

Sample	CathD (a) [ng mL^−1^]	CathD (b) [ng mL^−1^]	CathD (c) [ng mL^−1^]	CathD (d) [ng mL^−1^]
1 2 3 4 5 6 7 8 9	3.95 ± 0.32 4.49 ± 0.28	4.19 ± 0.29 4.95 ± 0.32	4.02 ± 0.11 4.22 ± 0.17	4.23 ± 0.13 4.81 ± 0.29
	3.07 ± 0.10 6.33 ± 0.41 7.49 ± 0.14 3.45 ± 0.12 3.56 ± 0.27 6.56 ± 0.33 3.45 ± 0.11	3.34 ± 0.21 6.87 ± 0.45 7.96 ± 0.31 3.97 ± 0.29 3.98 ± 0.17 6.99 ± 0.31 3.82 ± 0.22	3.11 ± 0.19 6.22 ± 0.60 7.36 ± 0.43 3.23 ± 0.19 3.30 ± 0.23 6.30 ± 0.34 3.33 ± 0.17	3.44 ± 0.23 6.79 ± 0.43 7.89 ± 0.61 3.78 ± 0.21 3.51 ± 0.31 6.79 ± 0.41 3.98 ± 0.21
Average	4.71 ± 1.64	5.12 ± 1.70	4.57 ± 1.62	5.02 ± 1.68

(a) pure gold chip and a two-paint separating polymer; (b) pure gold chip and adhesive foil as a separating polymer; (c) Ag/Au chip and a two-paint separating polymer; (d) Ag/Au chip and adhesive foil as separating polymer.

## Data Availability

Not applicable.
